# The first genotype determination of *Acanthamoeba* potential threat to human health, isolated from natural water reservoirs in Poland

**DOI:** 10.1007/s00436-014-3925-6

**Published:** 2014-04-27

**Authors:** Anna Lass, Beata Szostakowska, Alicja Idzińska, Lidia Chomicz

**Affiliations:** 1 Department of Tropical Parasitology, Medical University of Gdansk, 9b Powstania Styczniowego Str., 81-519 Gdynia, Poland; 2 Department of Medical Biology, Medical University of Warsaw, 73 Nowogrodzka Str., 02-018 Warsaw, Poland

**Keywords:** *Acanthamoeba*, PCR, Real-time PCR, Genotype, Environment, Water

## Abstract

Different species of amoebae belonging to the genus *Acanthamoeba* are widely distributed in many parts of the world and known as free-living organisms. Some strains of the protozoans may exist as parasites and cause risk to human health as causative agents of serious human diseases. Currently, in Poland, there is no sufficient information about the distribution of *Acanthamoeba* strains and their genotypes in the environment. Therefore, 20 environmental surface water samples were collected from different sites located at five water reservoirs in Gdynia, Sopot, and Gdańsk (northern Poland). The material was cultured to obtain *Acanthamoeba* isolates that were then specifically analyzed with both PCR and real-time PCR assays. Of the 20 samples examined, *Acanthamoeba* DNA was found in 13 samples tested with the use of real-time PCR; in 10 of them, DNA of the amoeba was also detected using PCR technique. The comparison with sequences available in the GenBank confirmed that the PCR products are fragments of *Acanthamoeba* 18S rRNA gene and that isolates represent T4 genotype, known as the most common strains related to AK cases. This is the first investigation in Poland describing *Acanthamoeba* detection in environmental water samples with molecular techniques and genotyping. The results indicate that surface water in Poland may be a source of acanthamoebic strains potentially pathogenic for humans.

## Introduction

Different species of the genus *Acanthamoeba* exist as free-living protists that are widely prevalent in natural and man-made environments. The amoebae were detected in soil, water, animals, fruits and vegetable, air, dust, hospital environment, contact lens solutions, and eyewash stations as well as isolated from human body surfaces and various human tissues and cavities (Larkin et al. 1990; Gray et al. 1995; Van Hamme et al. 2001; Chomicz et al. 2002; Schuster and Visvesvara 2004; Lorenzo-Morales et al. 2005b; Khan 2006; Carlesso et al. 2010; Costa et al. 2010; Stockman et al. 2011; Kao et al. 2012). The *Acanthamoeba* genus involves species that complete their life cycles in the outer environment. However, in predisposing circumstances, some strains of *Acanthamoeba* may be able to enter the human body from environmental sources and exist as parasites.

The high rate of specific antibodies (50–100 %) in healthy populations confirmed that humans are frequently exposed to *Acanthamoeba* (Alizadeh et al. 2001; Chappell et al. 2001; Brindley et al. 2009). Physiological and biochemical investigations carried out to determine pathogenic *Acanthamoeba* strains included assessment of temperature and osmo-tolerance as well as the secretion of proteolytic enzymes (Khan et al. 2000; Clarke and Niederkorn 2006; Chomicz et al. 2010). In immunocompetent individuals, infections with these amoebae are commonly asymptomatic and self-limited. However, some strains of *Acanthamoeba* species play a role in public health as causative agents of serious human diseases: the severe, usually fatal granulomatous amoebic encephalitis (GAE) recorded among immunocompromised patients and *Acanthamoebic* keratitis (AK), the non-opportunistic, vision-threatening corneal infection. The latter is found particularly in contact lens wearers, which make up 95 % of cases. Due to the popularity of contact lenses, that is considered an important risk factor in contracting corneal disease, incidents of an acute AK are increasingly recognized in various parts of the world, including Poland (Marciano-Cabral and Cabral 2003; Schuster and Visvesvara 2004; Wesołowska et al. 2006; Ibrahim et al. 2007; Visvesvara and Schuster 2008; Chomicz et al. 2012; Mahmoudi et al. 2012; Szaflik et al. 2012). Furthermore, the amoebae may transmit some yeast, viruses, and bacteria strains pathogenic for humans as well as oocysts of protozoan parasite *Cryptosporidium*; some of these microorganisms are able to not only survive but even proliferate within these amoebae (Gómez-Couso et al. 2007; Khan 2009; Scheid and Schwarzenberger 2011; Anacarso et al. 2012).

For years, an increasing number of isolates of *Acanthamoeba* species have been recognized and classified using morphological criteria, mainly cyst size and the number of arms within a single cyst. Amoebae belonging to the *Acanthamoeba* genus were placed into three morphological groups I, II, III (Pussard and Pons 1977; Page 1988; Khan 2009). Recently, this way of *Acanthamoeba* classification has begun to be seen as unreliable due to, among others, subtle differences in cyst features; non-morphological procedures were also used, including biochemical, immunological, and physiological criteria being inconclusive in the identification of *Acanthamoeba* species (Stratford and Griffiths 1978; Marciano-Cabral and Cabral 2003; Walochnik et al. 2002; Chan et al. 2010). The situation is changing with the recent development of specific and sensitive methods for classification of *Acanthamoeba* isolates at the molecular level. The new approach is based on genotype associations. Many PCR assays were designed to detect *Acanthamoeba* isolates in clinical and environmental samples with the high specificity and efficiency at the genus level (Vodkin et al. 1992; Howe et al. 1997; Pélandakis et al. 2000; Schroeder et al. 2001; Khan and Paget 2002; Khan et al. 2002; Pélandakis and Pernin 2002; Booton et al. 2005; Qvarnstrom et al. 2006). Also, real-time PCR was developed as a fast tool for differential identification of free-living amoebae (Qvarnstrom et al. 2006; Rivière et al. 2006). Recently, the most frequently used technique for the characterization *Acanthamoeba* isolates is phylogenic analysis based on the 18S rRNA gene sequence. The technique of molecular typing, used for the first time by Gast et al. (1996), indentified 17 genotypes (T1–T17) within the genus with a sequence divergence of >5 % (Stothard et al. 1998; Hewett et al. 2003; Khan 2009; Corsaro and Venditti 2010; Nuprasert et al. 2010). Of these, seven genotypes were found in patients with AK (T2, T3, T4, T5, T6, T11, T15) of which T4 was the highest frequency (Booton et al. 2005; Maghsood et al. 2005; Risler et al. 2013). At present, it is known that the majority of human diseases caused by infections with *Acanthamoeba* were associated with some isolates of the T4 genotype, i.e., more than 90 % of AK was linked with this genotype (Khan 2009). However, it is not clear why not all T4 isolates are human pathogens; among others, differences in virulence and in susceptibility to chemicals as well as complications associated with transmission by the amoebae potentially pathogenic microorganisms are investigated and discussed for this term (Maghsood et al. 2005; Khan 2009; Risler et al. 2013).

The number of clinical and environmental *Acanthamoeba* isolates is increasing as well as of studies concerning their prevalence in environmental samples using molecular techniques (Lorenzo-Morales et al. 2005a; Lorenzo-Morales et al. 2005b; Kawaguchi et al. 2009; Magliano et al. 2009; Niyyati et al. 2009; Chan et al. 2010; Rahdar et al. 2012). In Poland, only a few investigations regarding the occurrence of *Acanthamoeba* in environmental samples have been undertaken, based on the morphological criteria of identification of the amoebae and bioassays (Kasprzak and Mazur 1972; Befinger et al. 1986). No molecular techniques were used. Therefore, it is important to carry out such examinations in order to detect the presence of amoebae in the environment and identify their species and genotype.

The aim of our study was to estimate the occurrence of the *Acanthamoeba* spp. in chosen natural water reservoirs in Poland using molecular techniques. The investigations were also carried out to determine genotypes of the obtained environmental isolates in terms of their association with those genotypes indicating potential threat to human health.

## Material and methods

### Laboratory isolates

Different laboratory strains of free-living amoebae were used in the study. DNA isolated from these strains served as controls in molecular investigations. Four *Acanthamoeba* laboratory isolates *Acanthamoeba* *castellanii* Neff, *Acanthamoeba polyphaga*-98, *Acanthamoeba rhysodes*, and *Acanthamoeba astronyxis*-190 were cultivated axenically (Červa and Novak 1968) in the laboratory of the Department of Tropical Parasitology, Medical University of Gdansk. Isolates of *Naegleria*: *N. fowleri* (strains: V212, V004, V414, V087), *Naegleria italica*, *Naegleria lovaniensis*, and *Naegleria dunnebackei* as well as *Balamuthia mandrillaris* were obtained from the Centers for Disease Control and Prevention (CDC, Atlanta, GA, USA).

### Sampling and isolation of *Acanthamoeba* from the environmental material

Twenty surface water samples were collected in June and July 2006. Environmental samples of water with sediment in proportion 1:1 were taken from the Kacza River (*n* = 5), Morskie Oko Pond (*n* = 5), Wysockie Lake (*n* = 2), Jasień Lake (*n* = 3), and Głębockie Lake (*n* = 5) located in the area of three cities: Gdynia, Sopot, and Gdańsk (northern Poland). The samples were taken from two different sites at each location (Table 1). The material was placed in 100-ml sterile polypropylene bottles and transported to the laboratory. Each sample was deposited onto two non-nutrient agar plates seeded with *Aerobacter aerogenes*. The material was placed along the diameter of the plate as a 0.5-cm band. Next, one plate was incubated at room temperature and the second at 37 °C. The plates were monitored daily under a light microscope for *Acanthamoeba* growth. Then, agar blocks containing *Acanthamoeba* were cut out and inoculated on new non-nutrient agar plates seeded with *A. aerogenes* until a large number of amoebae were observed. Next, they were washed out with distilled water and the aliquots kept at 4 °C for further examinations.

**Table 1:** Results of molecular detection of *Acanthamoeba* in surface water samples taken from different water reservoirs localized in cities Gdynia, Gdańsk, and Sopot and comparison of obtained sequences of investigated *Acanthamoeba* isolates with the chosen *Acanthamoeba* strains showing 100 % homology of analyzed DNA fragment

Isolate no.	Sampling		Amoeba growth temp.	PCR	Real-time PCR	Length of obtained sequence, accession no.	Published 18S rRNA of *Acanthamoeba* spp. sequences available in the GenBank		
	Water reservoir	Site							
							Acanthamoeba species/strain	Accession no.	Origin of isolate
1	Kacza River, Gdynia	I	37	+	+	421 bp, KF924262	*A. castellanii* Neff	U07416	
							*A. castellanii*	GU001160	Marine sediment, England
							*A. castellanii*	KC164230	Compost, Switzerland
							*Acanthamoeba* sp.	GQ397475	Air conditioner scrap, Slovakia
							*Acanthamoeba* sp.	DQ087301	Ocular infection, France
2			37	+	+	421 bp, KF924263	Similar to 1		
3			37	+	+	421 bp, KF924264	Similar to 1		
4		II	37	+	+	415 bp, KF924265	*A. culbertsoni*	AF534139	Keratitis, non contact lens, India
							*A. lugdunensis*	AF260718	Nasal mucosa, Austria
							*A. rhysodes*	AF260720	Tap water, Austria
							*Acanthamoeba* sp.	GU320592	Coastal marine sediment, USA
							*Acanthamoeba* sp.	AY173008	Marine sediment, Korea
5			37	+	+	359 bp^a^			
6	Morskie Oko Pond, Sopot	III	37	+	+	421 bp, KF924266	*A. hatchetti* 2HH	AF260722	Keratitis, Korea
							*A. hatchetti* 2AX I	AF019060	Marine savage dump, USA
							*Acanthamoeba* sp.	AB425948	Rice field, Italy
							*Acanthamoeba* sp.	FJ422512	Keratitis, USA
7			Room temp.	+	+	306 bp^a^			
8		IV	37	+	+	421 bp, KF924267	Similar to 1		
9			37	+	+	416 bp, KF924268	*A. castellanii* Castellani	U07413	
							*A. castellanii*	KF318462	Brazil
							*Acanthamoeba* sp.	JX423603	Keratitis outbreak, USA
							*Acanthamoeba* sp.	JQ669659	Farm soil, USA
10			Room temp.	+	+	371 bp, KF924269	Similar to 9		
11	Wysockie Lake, Gdańsk	V	Room temp.	−	−				
12		VI	No growth	−	−				
13	Jasień Lake, Gdańsk	VII	37	−	−				
14			Room temp.	−	+	Not determined			
15		VIII	37	−	+	Not determined			
16	Głębockie Lake, Gdańsk	IX	37	−	−				
17			37	−	−				
18			Room temp.	−	−				
19		X	37	−	+	Not determined			
20			Room temp.	−	−				

+ indicates positive result of amplification; − indicates negative result of amplification

^a^Sequences do not embrace viable fragment DF3, belonging to genotype inconclusive, not deposited in GenBank

### Molecular detection of *Acanthamoeba*

#### DNA extraction

DNA extraction was performed using the commercial Genomic Mini Kit (A&A Biotechnology, Gdynia, Poland) according to the manufacturer’s instructions. Then, DNA was stored at −20 °C.

#### DNA amplification

For specific detection of *Acanthamoeba* DNA, two non-commercial methods PCR and real-time PCR developed by Schroeder et al. (2001) and Qvarnstrom et al. (2006), respectively, were used in this study as described below.

PCR reaction was undertaken with the use of the primers JDP1 (5′GGCCCAGATCGTTT ACCGTGAA3′) and JDP2 (5′TCTCACAAGCTGCTAGGGAGTCA3′) targeting ~450 bp fragment of *Acanthamoeba* 18S rRNA gene (Schroeder et al. 2001). The amplification reaction mixture consisted of 2.5 μl of 10×PCR buffer for RUN polymerase (A&A Biotechnology, Poland), 0.25 mM of each dNTP (Fermentas, Lithuania), 0.2 μM of each primer (Metabion, Germany) 0.75U of RUN polymerase (A&A Biotechnology, Poland), and 2 μl of template DNA in a 25-μl reaction volume. Amplifications were performed according to the original previously described conditions in a GeneAmp PCR System 9700 thermocycler (Applied Biosystems, USA). PCR products were analyzed using GelDoc-It Imaging Systems (UVP, USA) after electrophoresis on agarose gel (Sigma, St. Louis, Missouri) stained with ethidium bromide.

Real-time PCR was performed with the use of pair of primers AcantF900 (5′CCCAGATCGTTTACCGTGAA3′) and AcantR1100 (5′TAAATATTAATGCCCC CAACTATCC3′) targeting 180-bp fragment of the 18S rRNA gene and the fluorescently labeled TaqMan probe (5′Cy5 CTGCCACCGAATACATTAGCATGG BHQ33′) (Qvarnstrom et al. 2006). The amplification reaction mixture consisted of 12.5 μl of 2× Brilliant II QPCR Master Mix (Startagene, USA), 400 nM of each primer (Metabion, Germany), 80 nM of TaqMan probe (Metabion, Germany), and 2 μl of template DNA in a 25-μl reaction volume. Amplification was performed with an initial polymerase activation step (10 min at 95 °C), followed by 40 cycles of denaturation (15 s at 95 °C), and hybridization/extension (1 min at 63 °C) in an Mx3005P thermocycler (Stratagene, USA). PCR products were analyzed using MxPro QPCR Software. The cycle threshold (CT) value, determining the cycle number at which the reporter’s fluorescence exceeds the threshold value, was recorded. A sample was considered positive if the CT value was <40.

All PCR/real-time PCR experiments were performed with the inclusion of *Acanthamoeba* positive controls to ensure correct functionality of the reaction, *Naegleria*- and *Balamuthia*-positive controls to check specificity of the method as well as negative controls to ensure no contamination of the PCR components.

#### Sequencing

Direct sequencing was performed for eight obtained isolates. Before sequencing, PCR products were cleaned with the Cleanup Kit protocol (A&A Biotechnology, Poland). Cycle sequencing was performed with the use of the amplification primers and BigDye v.3.0 Terminator Cycle Sequencing Kit (Applied Biosystems, USA) in the GeneAmp PCR System 9700 thermocycler (Applied Biosystems, USA), in both orientations. The products of the sequencing reaction were cleaned using an ExTerminator Kit (A&A Biotechnology), denatured, and subjected to analysis on an automatic ABI PRISM 310 DNA Sequencer (Applied Biosystems) using standard procedures as described by the manufacturer. The sequences obtained were then analyzed, aligned, and compared with data from GenBank using GeneStudio Pro Software (GeneStudio, Inc., Suwanee, Georgia).

## Results

In 13 tested environmental samples, amoebae growth was observed at 37 °C, in six samples at room temperature, and in one sample, no amoebae growth was found (Table 1). The growth rate was graded by visual observation without enumeration of organisms.

The presence of *Acanthamoeba* DNA was recorded in 13 samples tested with the use of real-time PCR and in 10 samples using PCR. No product was found in DNA extracted from isolates of *Naegleria* and *Balamuthia*, which shows the specificity of chosen methods. The results confirmed that both methods chosen can be used to identify genus *Acanthamoeba*. However, real-time PCR was more sensitive than single-round PCR.

In order to determine the genotypes of *Acanthamoeba* isolates obtained from investigated environmental samples, sequencing of 10 PCR products was performed. In the case of the remaining three isolates (14, 15, and 19), that were positive only in real-time PCR, the amount of DNA was insufficient. The comparison of the obtained sequences with the *Acanthamoeba* sequences available in the GenBank confirmed that the detected PCR products were fragments of *Acanthamoeba* 18S rRNA gene. All sequenced isolates show 100 % homology with numbers of isolates belonging to the T4 genotype that is thought to be the cause most AK cases (Table 1). However, in the case of two isolates (samples 5 and 7), the sequences were shorter than in the other samples, and did not contain variable region (DF3 fragment). For this reason, it was impossible to exactly define their affiliation. Both of these isolates can be similar to each determined strain, or represent an additional one. Assuming the first option, four *Acanthamoeba* strains were defined. The variety of sequences was observed within one location, as well as between isolates obtained from different sampling sites (Table 1). It shows a high diversity within the *Acanthamoeba* genus.

Derived sequences containing DF3 fragment are deposited in GenBank under accession numbers KF924262–KF924269.

## Discussion

To our knowledge, it is the first investigation in Poland describing *Acanthamoeba* detection in environmental samples using rapid molecular detection methods. The results of our findings confirmed that free-living amoebae of *Acanthamoeba* genus are present in surface water in Poland. Moreover, all sequenced strains belong to the T4 genotype of *Acanthamoeba* which is known as the most common genotype related to AK cases. It indicates that surface water in Poland may be a source of acanthamoebic infections in humans, and more samples should be tested to estimate the prevalence of this organism in this and other environmental matrices including air and soil. It is known that the classification of *Acanthamoeba* spp. based on morphological criteria is insufficient, while their identification is not problematic using different PCR assays, including real-time PCR. In this study, we used PCR assay with primers JDP, as well as real-time PCR, both based on the 18S rRNA gene fragments. Amplification was successful in all tested water samples using real-time PCR and in three less using PCR, which confirmed the suitability of the methods to estimate the incidence of *Acanthamoeba* in environmental material. However, from the point of view of higher sensitivity, timesaving, as well as additional control of genus specificity by using a molecular probe, real-time PCR has an advantage.

Nevertheless, since Gast et al. (1996) developed a classification scheme based on nuclear rRNA gene sequence, it has become the most frequently used technique for the characterization of *Acanthamoeba* isolates. Phylogenetic analysis based on 18S rRNA gene sequence has enabled the identification of 17 genotypes (T1–T17) within the genus with a sequence divergence of >5 %. In the present study, the investigated isolates of *Acanthamoeba* were genotyped by DNA sequencing with the use of JDP primers enclosing a fragment of the 18S rRNA gene. Primers JDP delimit ~450-bp fragment of the sequence containing diagnostics fragment 3 (DF3) which shows high variability within the genotypes of *Acanthamoeba*. A DF3 sequence was often used as a target in phylogenetic analysis in order to classify isolates to particular sequence types. However, some authors claim that this region is insufficient to discriminate isolates closely related T3, T4, and T11 genotypes (Schroeder et al. 2001; Risler et al. 2013). According to the authors, in some cases, *Acanthamoeba* isolates with the same DF3 sequence were not necessarily identical. For this reason, other variable regions of the 18S rRNA gene than DF3 should be used for correctly typing the isolates. This will be taken under consideration in our next study.

The majority of AK cases worldwide are connected with the genotype T4 and rarely with T2, T3, T6, and T11 (Walochnik et al. 2000; Khan et al. 2002; Maghsood et al. 2005; Ledee et al. 2009; Risler et al. 2013). It is not clear yet if the high isolation rate of the T4 genotype related to AK cases worldwide may be due to their greater prevalence in the environment, greater virulence or both (Khan 2006). However, many previous studies similarly showed that the T4 genotype is the most prevalent in the environment (Maghsood et al. 2005; Magliano et al. 2009; Niyyati et al. 2009; Rahdar et al. 2012).

*Acanthamoeba* spp. were found in different water samples worldwide. For instance, they were present in tap water in Brazil (Magliano et al. 2009); rivers, waterfall, and swimming pools in Iran (Maghsood et al. 2005; Niyyati et al. 2009; Rahdar et al. 2012); rivers and water treatment plants in Japan (Edagawa et al. 2009; Kawaguchi et al. 2009); and rivers, springs, wells, and water tanks in Nicaragua (Leiva et al. 2008). In this study, we investigated surface water samples taken from rivers and pond located at the area of three cities: Gdynia, Sopot, and Gdańsk in northern Poland. We found *Acanthamoeba* isolates in 65 % of samples tested. The results of sequencing the isolates showed 100 % similarity to sequences of isolates representing the T4 genotype deposited in GenBank. The differences noticed between the two locations (river in Gdynia and pond in Sopot) as well as within one location among two remote sampling sites confirm the heterogeneous nature of *Acanthamoeba*.

Human infections caused by *Acanthamoeba* remain poorly investigated in Poland. However, cases of acanthamoebic keratitis and studies with clinical isolates have been reported, and isolated strains were classified as T4 genotypes (Wesołowska et al. 2006; Szaflik et al. 2012). Environmental studies were rarely performed, mainly in the 1980s using morphological and physiological criteria. Among others, Befinger et al. (1986) found *Naegleria* and *Acanthamoeba* species in Lake Żarnowieckie and the Piaśnica River (northern Poland). Also, around Poznań (a city in western Poland), some lakes, a river and a canal used as recreational resorts, showed a common presence of *Limax* group amoebae (Kasprzak and Mazur 1972). Nowadays, the diversity and pathogenic potential of *Acanthamoeba* spp. and other free-living amoebae from Polish water reservoirs as well as other environmental matrices remain unknown.

This is the first report presenting results of our molecular studies on occurrence of *Acanthamoeba* genotypes in environment material. The results provide evidence that *Acanthamoeba* strains probably belonging to the T4 genotype, believed to be associated with AK cases, are present in the investigated water reservoirs, in northern Poland. For this reason, a larger number of environmental samples should be tested, taking into account different locations and water reservoir types, as well as studies relating to the diversity of *Acanthamoeba* strains and their prevalence and pathogenic potential.
